# Retraction: Carulli, L. et al. The OMICs Window into Nonalcoholic Fatty Liver Disease (NAFLD). *Metabolites* 2019, *9*(2), 25

**DOI:** 10.3390/metabo9040070

**Published:** 2019-04-11

**Authors:** Lucia Carulli, Giulia Zanca, Filippo Schepis, Erica Villa, Metabolites Editorial Office

**Affiliations:** 1 Division of Gastroenterology, Department of Medical Specialties, Azienda Ospedaliero-Universitaria and University of Modena and University of Modena and Reggio Emilia, 41124 Modena, Italy; lucia.carulli@unimore.it (L.C.); 165382@studenti.unimore.it (G.Z.); filippo.schepis@unimore.it (F.S.); 2 St. Alban-Anlage 66, 4052 Basel, Switzerland

As the authors of the title paper [1], it is with great regret that we inform the readership of *Metabolites* that we have asked the journal’s publisher, MDPI, to retract the paper from the scientific literature. Due to human error, we included contents similar to article [2], which has already been published by Pirola et al. We apologize to the readership of *Metabolites* and to the authors of [2] for any inconvenience caused.

MDPI is a member of the Committee on Publication Ethics (COPE) and takes the responsibility to enforce strict ethical policies and standards very seriously. To ensure the integrity of the publication record, [1] is retracted and shall be marked accordingly.

## References

[1] Carulli L., Zanca G., Schepis F., Villa E. (2019). The OMICs Window into Nonalcoholic Fatty Liver Disease (NAFLD). Metabolites.

[2] Pirola C.J., Sookoian S. (2018). Multiomics biomarkers for the prediction of nonalcoholic fatty liver disease severity. World J. Gastroenterol..

